# Freshwater colonization drives divergent reproductive strategies in shrimps of the genus *Palaemon* (Decapoda: Palaemonidae)

**DOI:** 10.1007/s00114-026-02092-5

**Published:** 2026-03-27

**Authors:** Caio S. Nogueira, Sara C. Gasparotto, Matheus Sene, Lucas Oliveira-Rogeri, Isabela R. R. Moraes, Laura S. López Greco, Fernando J. Zara, Lucas R. P. Paschoal

**Affiliations:** 1 Invertebrate Morphology Laboratory (IML), Department of Biology, School of Agriculture and Veterinary Sciences (FCAV), São Paulo State University (UNESP), Jaboticabal, São Paulo, Brazil; 2 Faculty of Sciences, Multidisciplinary Unit for Teaching and Research (UMDI), National Autonomous University of Mexico (UNAM), Puerto de Abrigo, Sisal, Hunucmá, Yucatán, Mexico; 3 Laboratory of Biology of Marine and Freshwater Shrimps (LABCAM), Department of Biological Sciences, Faculty of Sciences, São Paulo State University (UNESP), Bauru, São Paulo, Brazil; 4 Laboratory of Bioecology and Systematics of Crustaceans (LBSC), Department of Biology, Faculty of Philosophy, Science and Letters at Ribeirão Preto (FFCLRP), University of São Paulo (USP), Ribeirão Preto, SP Brazil; 5 Laboratory of Carcinology, Department of Zoology, Institute of Biosciences, Federal University of Rio Grande do Sul (UFRGS), Porto Alegre, Rio Grande do Sul Brazil; 6 Facultad de Ciencias Exactas y Naturales, Departamento de Biodiversidad y Biología Experimental, Laboratorio de Biología de la Reproducción, Crecimiento y Nutrición de Crustáceos Decápodos, Universidad de Buenos Aires, Ciudad Universitaria, Buenos Aires, C1428EGA Argentina; 7 CONICET – Universidad de Buenos Aires, Instituto de Biodiversidad y Biología Experimental y Aplicada (IBBEA), Buenos Aires, Argentina

**Keywords:** Crustaceans, Fecundity, Freshwaterization, Reproductive traits, Spermatozoa

## Abstract

Freshwater colonization is associated with profound shifts in the reproductive traits of invertebrates, and these phenomena are still rarely investigated in an integrated framework across closely related species. In this study, we examined three species of shrimps of the genus *Palaemon*, each occupying distinct habitat types, to identify how the transition to freshwater habitats shapes reproductive investment strategies. For each species, we quantified multiple reproductive traits, including fecundity, spermatozoa count, embryo and spermatozoa dimensions, reproductive output, per-offspring investment, and investment in weaponry. Our results revealed parallel patterns between sexes: *P. northropi*, the marine species, exhibited the highest fecundity and spermatozoa production, whereas *P. yuna*, the freshwater species, displayed the lowest values for both traits. Embryo and gamete sizes varied coordinately, with *P. yuna* producing larger embryos and spermatozoa, consistent with the abbreviated larval development typical of freshwater species. Per-offspring investment in *P. yuna* was up to approximately 16 times higher than in the other species, reflecting the substantial energetic costs associated with this developmental strategy. Overall, we show that freshwater colonization imposes adaptive selective pressures that reshape multiple components of reproduction in an integrated way, affecting not only females but also males. These findings demonstrate that coordinated adjustments in the number, size, and energetic cost of gametes represent key evolutionary responses underpinning reproductive success in freshwater environments.

## Introduction

The transition between marine, estuarine, and freshwater environments represents one of the major ecological axes driving diversification in aquatic organisms. Variations in salinity, environmental stability, and resource availability impose distinct selective pressures, often reflected in adjustments to reproductive strategies (Winemiller and Rose 1992; Anger 2001, 2006; Vogt 2013). Across multiple taxa, the colonization of lower-salinity environments is frequently associated with increased energetic investment per offspring, evidenced by the production of larger but fewer embryos. This strategy results in a more prolonged embryonic development, ultimately leading to the hatching of individuals at more advanced developmental stages (Winemiller and Rose 1992; Anger 2001; Vogt 2013; Pantaleão et al. 2025).

This pattern is widely observed among crustaceans, in which freshwater species generally produce larger, lecithotrophic eggs and exhibit abbreviated or even direct development when compared to most marine or estuarine species (Vogt 2013). The extension of embryonic development and the subsequent abbreviation of larval stages have evolved independently across several groups, including ostracods, cladocerans, leptostracans, peracarids, and pleocyemate decapods (Vogt 2013). In decapods specifically, nearly half of the infraorders include species that have colonized estuarine or freshwater environments (e.g., Dendrobranchiata, Caridea, Astacidea, Anomura, and Brachyura; Bond-Buckup et al. 2008; Vogt 2013; Cumberlidge 2016; Crandall and De Grave 2017; Bauer 2023). Among pleocyemates, several adaptations are strongly associated with life in strictly freshwater habitats, such as prolonged embryonic development, the reduction or complete loss of the planktonic larval stage, and, in some groups, extended parental care (Vogt 2013).

Abbreviated or direct larval development is predominant among pleocyemate crustaceans that have colonized freshwater environments (Vogt 2013; Bauer 2023). However, this condition also occurs in marine organisms, typically associated with specialized lifestyles, such as symbiosis, or with the occupation of stressful habitats characterized by low resource availability (Vogt 2013). Examples include several caridean shrimp species of the genus *Synalpheus* Spence Bate, 1888, which inhabit sponges and may exhibit abbreviated or direct larval development (Duffy and Macdonald 2009), as well as certain deep-sea crangonid and pandalid shrimps that exhibit a reduced number of larval stage**s** (King and Butler 1985; Sedova and Grigoriev 2018; Fujita et al. 2021). Nonetheless, among marine shrimps, only a small fraction displays such adaptations, with the predominant pattern being extended larval development, typically characterized by multiple (more than three) zoeal stages (Pike and Williamson 1964; Haynes 1980; Yang et al. 2009; Santos et al. 2025).

Among decapods, the simplest form of parental care is the incubation of embryos until larval hatching (Thiel 2000; Vogt 2013; Palaoro and Thiel 2020), a trait observed in some dendrobranchiate shrimps and in all pleocyemates (Vogt 2013; Bauer 2023). As in the embryonic and post-embryonic stages, prolonged parental care is more common among freshwater species, such as brachyuran crabs, aeglid crabs, and crayfish, in which juveniles remain attached to the female’s pleon for several days after hatching (Vogt 2013). However, exceptions exist, and similar forms of prolonged parental care have also been reported in marine species, including some caridean shrimps, brachyuran crabs, and anomurans (Wear 1967; Haynes 1985; Bolaños et al. 2004; Calado et al. 2006; Guay et al. 2011). These findings indicate that parental-care strategies, as well as variations in embryonic and post-embryonic development, may have evolved independently in multiple decapod lineages, reflecting convergent evolutionary responses to similar ecological pressures.

Taken together, these observations highlight pleocyemate crustaceans as excellent model systems for investigating how ecological selective pressures and the colonization of freshwater environments can shape reproductive traits. This approach becomes particularly valuable in groups that include closely related species distributed across different habitat types (e.g., marine, estuarine, and freshwater), such as shrimps of the genus *Macrobrachium* Spence Bate, 1888, with estuarine and freshwater representatives, and *Palaemon* Weber, 1795, with species occurring in all three kinds of environments (Ashelby et al. 2012; De Grave and Ashelby 2013; Vogt 2013; Carvalho et al. 2017; Pantaleão et al. 2025). Species from these two genera exhibit two distinct patterns of larval development, namely Extended Larval Development (ELD) and Abbreviated Larval Development (ALD), which reflect contrasting reproductive strategies and different levels of energetic investment per offspring (Magalhães and Walker 1988; Vogt 2013; Bauer 2023; Pantaleão et al. 2025).

Currently, ten species of the genus *Palaemon* are recognized in Brazil (Terossi and Cardoso 2024), including five strictly freshwater species, one estuarine species, and four exclusively marine species (Carvalho et al. 2017, 2020). All freshwater species are endemic to the Brazilian Amazon region, whereas the remaining species occur along different locations of the Brazilian coast (Carvalho et al. 2017, 2020). Considering the variation in reproductive effort that may exist among these species, the present study aimed to conduct a comprehensive analysis of multiple reproductive traits in three *Palaemon* species from Brazil: *P. northropi* (Rankin, 1898), an exclusively marine species, *P. pandaliformis* (Stimpson, 1871), an amphidromous species that primarily inhabits estuarine environments, and *P. yuna* Carvalho et al. 2014; a freshwater species from the Amazon.

Multifactorial analyses of reproductive traits in caridean shrimps remain poorly explored in the literature, particularly within the genus *Palaemon*, where research has primarily focused on female reproductive performance. Most studies emphasize fecundity and reproductive output based on morphometric data and the mass of embryos, ovaries, and adult females (Kim and Hong 2004; Mortari et al. 2009; Janas and Mańkucka 2010; Paschoal et al. 2016). The incorporation of information on male reproductive traits underscores the importance of conducting this type of integrative approach not only for the model species addressed in the present study, but also for broader comparative investigations across crustaceans. Moreover, examining the energetic investment in the development of weapons such as chelipeds, structures directly involved in combat, mate searching, and other agonistic behaviors, reveals important interactions with reproductive investment in both males and females (Hughes et al. 2014; Nogueira et al. 2022; Paschoal and Zara 2022). This integrated perspective allows the construction of a comprehensive multifactorial framework, strengthening adaptive and evolutionary interpretations of the reproductive strategies of the species examined here.

Based on these premises, we tested the hypothesis that freshwater colonization is associated with coordinated shifts in reproductive investment in *Palaemon* species, fecundity, embryo size, reproductive output, investment per offspring, investment in weapons, and spermatozoa number and size. Specifically, we predicted that species inhabiting freshwater environments would exhibit lower fecundity and spermatozoa production, but larger embryos and spermatozoa, higher per-offspring investment, and reduced relative investment in weaponry when compared to estuarine and marine congeners. These parameters were compared and discussed in relation to each species’ environmental origin (marine, estuarine, or freshwater) to understand how the colonization of freshwater habitats may shape reproductive characteristics within this genus.

## Materials and methods

### Model species

*Palaemon northropi* is a marine species widely distributed throughout the Americas and commonly found along the Brazilian coast, from the northeastern to the southern regions (Carvalho et al. 2020). These organisms are typically encountered during low tide in tide pools along rocky shores. Previous studies have investigated some of its reproductive traits, exclusively in females, including reproductive period, fecundity, embryo size, and larval development. It is known to produce numerous small embryos, a characteristic of species with extended larval development (Moura et al. 1990; Anger and Moreira 1998; Barros-Alves et al. 2019).

*Palaemon pandaliformis* is an estuarine species with a broad distribution across Brazil, ranging from the northern to the southern estuarine regions (Carvalho et al. 2020). These organisms are typically associated with the roots of mangrove vegetation. This species has also been the focus of reproductive studies, and it is well established that it presents continuous reproduction throughout the year and produces numerous small embryos typical of extended larval development (Gamba et al. 1998; Anger and Moreira 1998; Mortari et al. 2009; Paschoal et al. 2016).

*Palaemon yuna* is a strictly freshwater species, with a distribution restricted to the interior of the Amazon basin. Initially, its records were limited to the Negro River basin in the state of Amazonas, but its presence was later confirmed in the Branco River basin, in the state of Roraima (Carvalho et al. 2014; Santos et al. 2018). Although no studies have specifically addressed the biology of this species, it is hypothesized to exhibit reproductive traits similar to those of sympatric congeners, such as *Palaemon ivonicus* (Holthuis, 1950), and to possess characteristics associated with abbreviated larval development (Magalhães and Walker 1988).

### Specimen sampling

*Palaemon northropi* and *P. pandaliformis* were collected at different sites along the southeastern coast of Brazil, specifically in the municipality of Ubatuba, São Paulo State, in April 2024. Specimens of *P. northropi* were collected at Praia do Lamberto (23°30’03.5"S, 45°07’07.6"W), located within the facilities of the Oceanographic Institute, University of São Paulo (IO-USP). This species inhabits the rocky intertidal zone, and sampling was conducted at night during low tide, when small tide pools become exposed. Individuals of *P. pandaliformis* were collected in the mangrove of the Escuro River (23°29’23.9"S, 45°09’53.3"W) during the morning, also at low tide.

Specimens of *P. yuna* were collected in northern Brazil, in the municipality of Manaus, Amazonas State, in January 2024. Sampling was conducted at six sites distributed across two nearby freshwater bodies (approximately 10 km apart), both draining into the Negro River. Three sites were located in floodplain areas of Lake Tupé (Site 1 = 3°01’35.3”S, 60°15’55.4”W; Site 2 = 3°01’48.3”S, 60°16’07.1”W; Site 3 = 3°02’14.1”S, 60°14’56.6”W), and three others in the Tarumã-Mirim River (Site 1 = 3°00’58.5”S, 60°11’04.0”W; Site 2 = 3°01’05.0”S, 60°11’01.8”W; Site 3 = 3°01’12.1”S, 60°10’06.3”W). Collections were carried out throughout the morning and afternoon hours.

Shrimp sampling in this study consisted of a one-hour active search conducted by four collectors at each sampling site within each locality. For *P. northropi*, sampling was performed using handheld aquarium nets (30 cm total length, 10 cm wide net basket). Specimens were collected from tidal pools or from rocks or macroalgae, particularly *Sargassum* spp.. For *P. pandaliformis* and *P. yuna*, active searches employed circular sieves with 3-mm mesh, operated within submerged vegetation and among emergent or floating macrophytes. Each sieve was submerged and swept through the vegetation to capture associated organisms. After collection, shrimps were placed in containers filled with water from the sampling site and transported to the laboratory. Individuals were euthanized by chilling (-20 °C) and preserved in 95% ethanol.

Specimens were identified based on the diagnostic characters described by Carvalho et al. (2020). Sex determination was conducted through morphological examination of the second pair of pleopods, considering the presence of the *appendix masculina* on the endopod (males) or its absence (females) (Bauer 2023). Ovigerous females were recognized by the presence of the embryo masses attached to the pleopods. To characterize body size in females and males of the three species, shrimps were measured for carapace length (CL), defined as the distance between the posterior margin of the ocular orbit and the posterior margin of the carapace. Measurements were taken using a digital caliper with a 0.01 mm precision.

### Analyses of female and male reproductive traits

Before analysis, all datasets were tested for normality using the Shapiro-Wilk test (α = 0.05). According to the results, parametric or non-parametric analyses were applied as appropriate. All statistical procedures were performed in PAST version 5.3 (Hammer et al. 2001).

### Morphometric characterization and sexual dimorphism

Sexual dimorphism in body size was assessed for all analyzed species. The CL values were compared between males and females within each species using a Student’s t-test (for normally distributed data) or a Mann-Whitney U test (for non-normal data). Subsequently, potential morphometric differences between females and males of each species were evaluated using a Kruskal-Wallis test followed by Dunn’s post hoc test. For these analyses, a total of 30 males and 30 females from each species were included.

### Fecundity and spermatozoa count

Mean fecundity for each species was estimated by directly counting the total number of embryos carried by each ovigerous female in the brood chamber. A total of 30 females per species were analyzed, except for *P. yuna*, for which only six ovigerous females were obtained. Embryos were carefully removed from the pleon using a pipette, and the brood chamber was rinsed with 95% ethanol to release embryos that had adhered to the pleopods. The embryo masses of each female were kept separate and subsequently counted individually. All ovigerous females analyzed carried embryos at an early developmental stage (i.e., completely yolk-filled and lacking eye spots).

For spermatozoa count analysis, five males from each species were used, following the protocol proposed by Paschoal and Zara (2018) for caridean shrimps, with minor adaptations due to the low spermatozoa concentration observed in the species analyzed in the present study. Individuals were randomly selected, and the male reproductive system was dissected and removed. The testes were then separated from the vas deferens, and one vas deferens (VD) was randomly chosen for analysis. This VD was sectioned at the beginning of the dilation that marks the transition between the median and distal regions, a morphological pattern characteristic of Palaemonidae (Poljaroen et al. 2010; Paschoal and Zara 2018; Nogueira et al. 2023, 2025). Thus, the distal portion of the VD was standardized as the reference region for spermatozoa counting in all individuals analyzed.

The distal region of the VD was dissociated in a solution containing 9 µL of distilled water and 1 µL of methylene blue. Then, 1 µL of this solution was carefully pipetted into the central region of a Neubauer chamber for spermatozoa counting under a light microscope (at 20× magnification). Sample preparation, randomization, and the estimation method followed the procedures described by Paschoal and Zara (2018). In this study, spermatozoa count was expressed as the number of spermatozoa per microliter (spz/µL).

The relationship between the size of ovigerous females and fecundity was evaluated using Spearman’s correlation between CL and the total number of embryos per female. To compare mean fecundity among the three species, a Kruskal-Wallis test was applied, followed by Dunn’s post hoc test. Additionally, to reduce the effect of female size on fecundity, the fecundity index (FI) was employed, as proposed by Anger (1995), where FI = number of embryos / CL. FI values among species were compared using the same statistical procedure applied to mean fecundity.

The comparison of mean spermatozoa counts among males of the three species was performed using an analysis of variance (ANOVA), followed by Tukey’s post hoc test. In this case, no correction related to body size was applied, since in shrimps, male size (CL) does not exert a significant influence on the number of spermatozoa produced (Paschoal and Zara 2018; Nogueira et al. 2023).

### Embryos and spermatozoa size

After embryo counting (i.e., fecundity analysis), 15 embryos from each ovigerous female were randomly selected and measured for their major (L) and minor (S) axes to estimate the mean embryo volume for each species. Measurements were taken from images obtained under a Leica^®^ MZ stereomicroscope using the Leica Application Suite software. Embryo volume (V) was calculated according to the formula V = (π × L × S²) / 6, as proposed by Odinetz-Collart and Rabelo (1996).

**Fig. 1:**
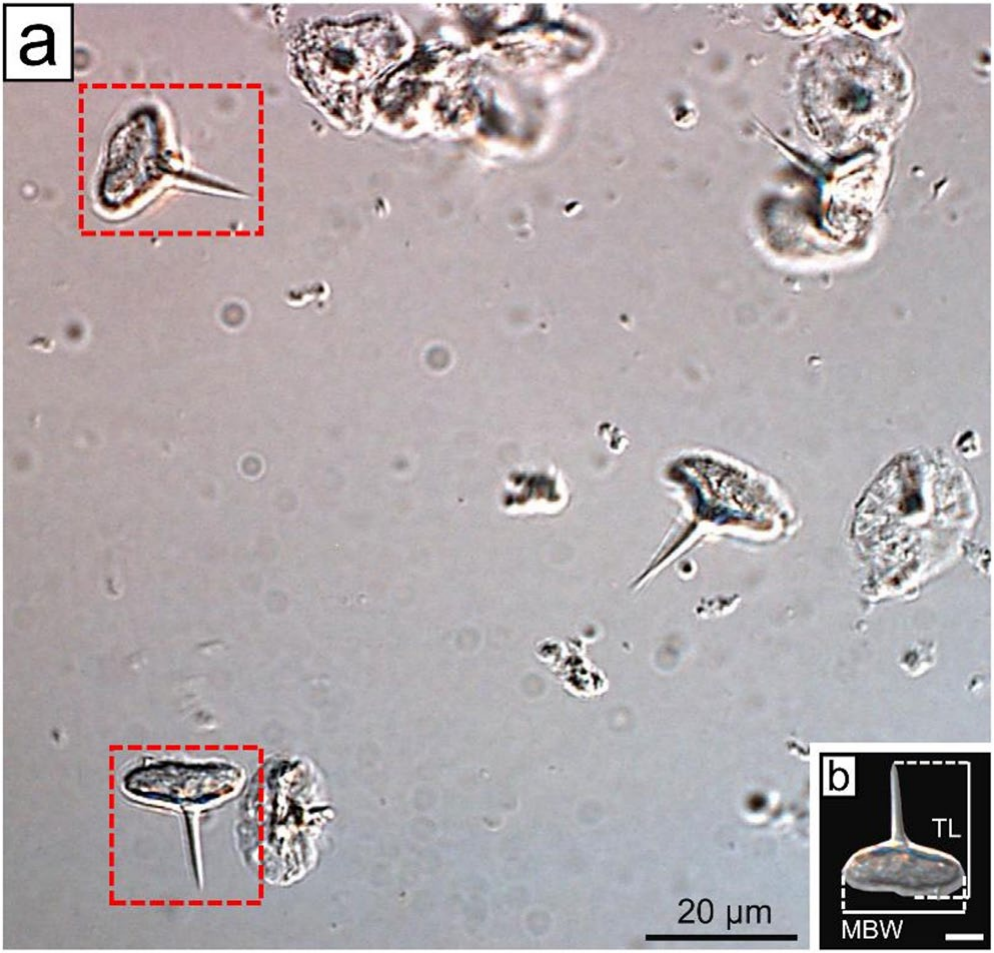
Differential interference contrast (DIC) microscopy. **a** Micrograph showing spermatozoa of *Palaemon pandaliformis*. The spermatozoa highlighted in red are positioned according to the standard orientation used for measurements. **b** Spermatozoon of *P. pandaliformis* with indication of the measured variables. Scale bar in B corresponds to 5 μm. MBW: main body width; TL: total spermatozoa length

For the morphometric analysis of spermatozoa, fragments of the VD from five males of each species were used. These fragments were obtained from the same individuals employed for the spermatozoa count. Each fragment was macerated in 5 mL of phosphate buffer solution, and a 100 µL aliquot of the resulting suspension was dropped onto a microscope slide and covered with a coverslip (Nogueira et al. 2025). The slides were examined under a Zeiss Axio Imager Z2 optical microscope equipped with differential interference contrast (DIC). Spermatozoa measurements were taken using the Zeiss AxioVision software to compare morphometric characteristics among species. Different cellular regions were assessed, including total spermatozoa length (TL) and the main body width (MBW) of the spermatozoon (Fig. 1A, B). For each male, a total of 20 spermatozoa were measured.

The comparison of embryo volume among females and spermatozoa size among males of the three species was performed using a Kruskal-Wallis test, followed by Dunn’s post hoc test. In addition, Pearson’s correlation coefficient was used to assess the association between embryo volume in females and spermatozoa size in males for each species of *Palaemon*.

### Reproductive output, per-offspring investment, and weapon investment

To calculate reproductive output, per-offspring investment, and weapon investment (for both females and males), we used the wet weights of the body, chelipeds, and embryo masses. Excess water from the body, chelipeds, and embryo masses was removed using absorbent paper. All structures were then weighed on an analytical balance with a precision of 0.0001 g. The same ovigerous females previously analyzed were used, along with 30 males of each species for the weapon-investment analyses. For the assessment of weapon investment in *P. yuna* females, the six ovigerous females were included, and an additional 24 non-ovigerous females were added to reach the required sample size for the analyses.

Reproductive output (RO) was estimated using the formula RO = (embryo mass weight / female body weight) × 100, and expressed as a percentage. Before calculating per-offspring investment (OI), the average weight of a single embryo (AWE) was determined for each ovigerous female. This value was obtained individually by dividing the total embryo mass weight by the number of embryos carried by each female. Subsequently, OI was calculated using the formula OI = (AWE / female body weight) × 100, also expressed as a percentage.

Finally, weapon investment (WI) was calculated as the relative contribution of cheliped mass to body mass. Thus, WI was calculated as WI = (cheliped weight / shrimp body weight) × 100. The right cheliped was standardized for this analysis. Only one cheliped was used because the three analyzed species do not exhibit heterochely. Reproductive output, per-offspring investment, and weapon investment values were compared between the sexes of each species using the Kruskal-Wallis test, followed by Dunn’s post hoc test.

## Results

### Morphometric characterization and sexual dimorphism

**Fig. 2:**
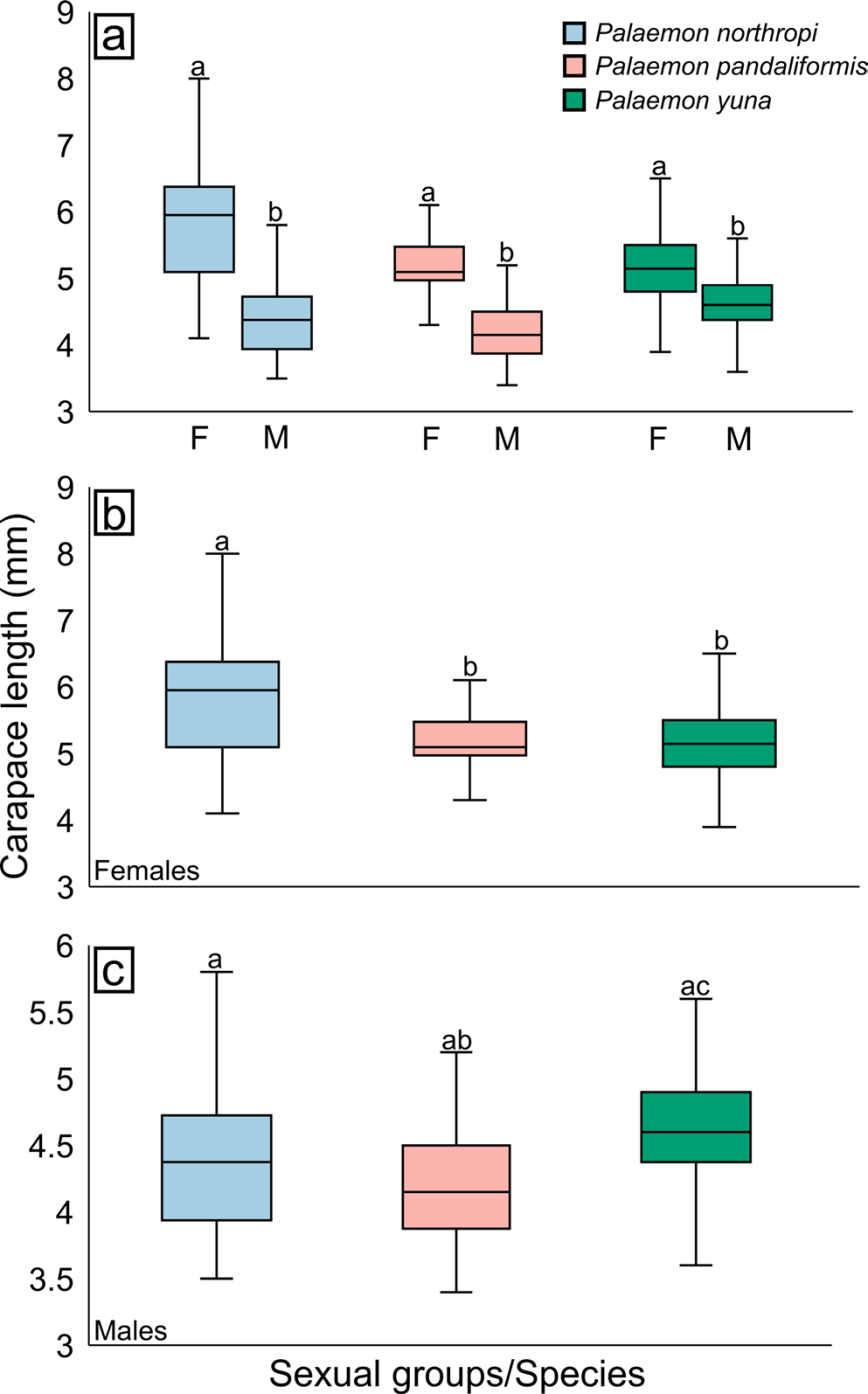
Body size variation and sexual dimorphism in *Palaemon* spp. **a** Sexual dimorphism in the three species, showing that females are larger in all comparisons. **b** Body size variation among females of the three species; females of *P. northropi* are the largest. **c** Body size variation among males of the three species. On average, males of *P. yuna* are the largest, although not significantly different from the second largest species (*P. northropi*). Different letters above the boxplots indicate significant differences among the compared groups (*p* < 0.05). F: Females; M: Males

Females and males of *P. northropi* exhibited mean CL values of 5.93 ± 1.13 mm (min–max: 4.1–9.3) and 4.40 ± 0.57 mm (3.5–5.8), respectively. In *P. pandaliformis*, mean CL was 5.18 ± 0.58 mm (4.2–6.1) for females and 4.24 ± 0.59 mm (3.4–6.4) for males. For *P. yuna*, females had a mean CL of 5.13 ± 0.56 mm (4.2–6.5), while males measured 4.59 ± 0.44 mm (3.6–5.6) (Fig. 2A; Table 1).

**Table 1:** Description of the variation in the morphological and reproductive traits analyzed among *Palaemon* spp. The measurement unit for each trait is indicated in parentheses in the column header. Different letters in the same line indicate significant differences among the compared groups (*p* < 0.05). MBW: main body width; TL: spermatozoa total length

Morphological/ Sexual trait	Species		
	*Palaemon northropi*	*Palaemon pandaliformis*	*Palaemon yuna*
Caparace length (♀ and ♂; mm)	5.93 ± 1.13^a^ and 4.4 ± 0.57^a^	5.18 ± 0.58^b^ and 4.24 ± 0.59^ab^	5.13 ± 0.56^b^ and 4.59 ± 0.44^ac^
Fecundity (embryos)	257 ± 133^a^	136 ± 43^b^	21 ± 6^c^
Fecundity index (embryos)	41 ± 17^a^	26 ± 6^b^	4 ± 1^c^
Spermatozoa count (spz/µL)	805 ± 208^a^	499 ± 47^b^	236 ± 41^c^
Embryo size (mm³)	0.11 ± 0.03^a^	0.14 ± 0.02^b^	0.86 ± 0.09^c^
Spermatozoa size (TL and MBW; µm)	14.35 ± 1.31^a^ and 12.65 ± 1.79^a^	20.03 ± 1.56^b^ and 14.53 ± 1.31^ab^	23.8 ± 1.6^c^ and 21.91 ± 2.7^c^
Reproductive output (%)	6.34 ± 2.49^a^	10.01 ± 2.3^b^	9.5 ± 1.93^b^
Per-offspring investment (%)	0.03 ± 0.01^a^	0.08 ± 0.03^b^	0.48 ± 0.08^c^
Weaponry investment (♀ and ♂; %)	0.64 ± 0.29^a^ and 0.41 ± 0.12^a^	0.43 ± 0.10^b^ and 0.20 ± 0.07^b^	0.34 ± 0.10^c^ and 0.28 ± 0.07^c^

Significant differences were detected between sexes in all species analyzed, with females being consistently larger than males [*P. northropi* – Mann-Whitney test, U = 74, *p* < 0.001; *P. pandaliformis* – t-test, t = 6.27, *p* < 0.001; and *P. yuna* – t-test, t = 4.15, *p* < 0.001].

Additionally, significant differences were also detected when comparing the size of species for both females (Kruskal-Wallis test, H = 11.1, *p* = 0.003) and males (Kruskal-Wallis test, H = 9.85, *p* = 0.007). Among females, *P. northropi* differed significantly from *P. pandaliformis* (Dunn’s test, *p* = 0.006) and from *P. yuna* (Dunn’s test, *p* = 0.003), whereas no differences were detected between *P. pandaliformis* and *P. yuna* (Dunn’s test, *p* = 0.79) (Fig. 2B). Among males, a significant difference was observed between *P. pandaliformis* and *P. yuna* (Dunn’s test, *p* = 0.002), but not between *P. northropi* and *P. pandaliformis* (Dunn’s test, *p* = 0.16), nor between *P. northropi* and *P. yuna* (Dunn’s test, *p* = 0.08) (Fig. 2C).

### Fecundity and spermatozoa count

A strong positive relationship between CL and fecundity was detected in all three species, with larger females brooding a greater number of embryos (*P. northropi* – ⍴ = 0.74, *p* = 0.003; *P. pandaliformis* – ⍴ = 0.72, *p* < 0.001; *P. yuna* – ⍴ = 0.64, *p* = 0.02).

**Fig. 3:**
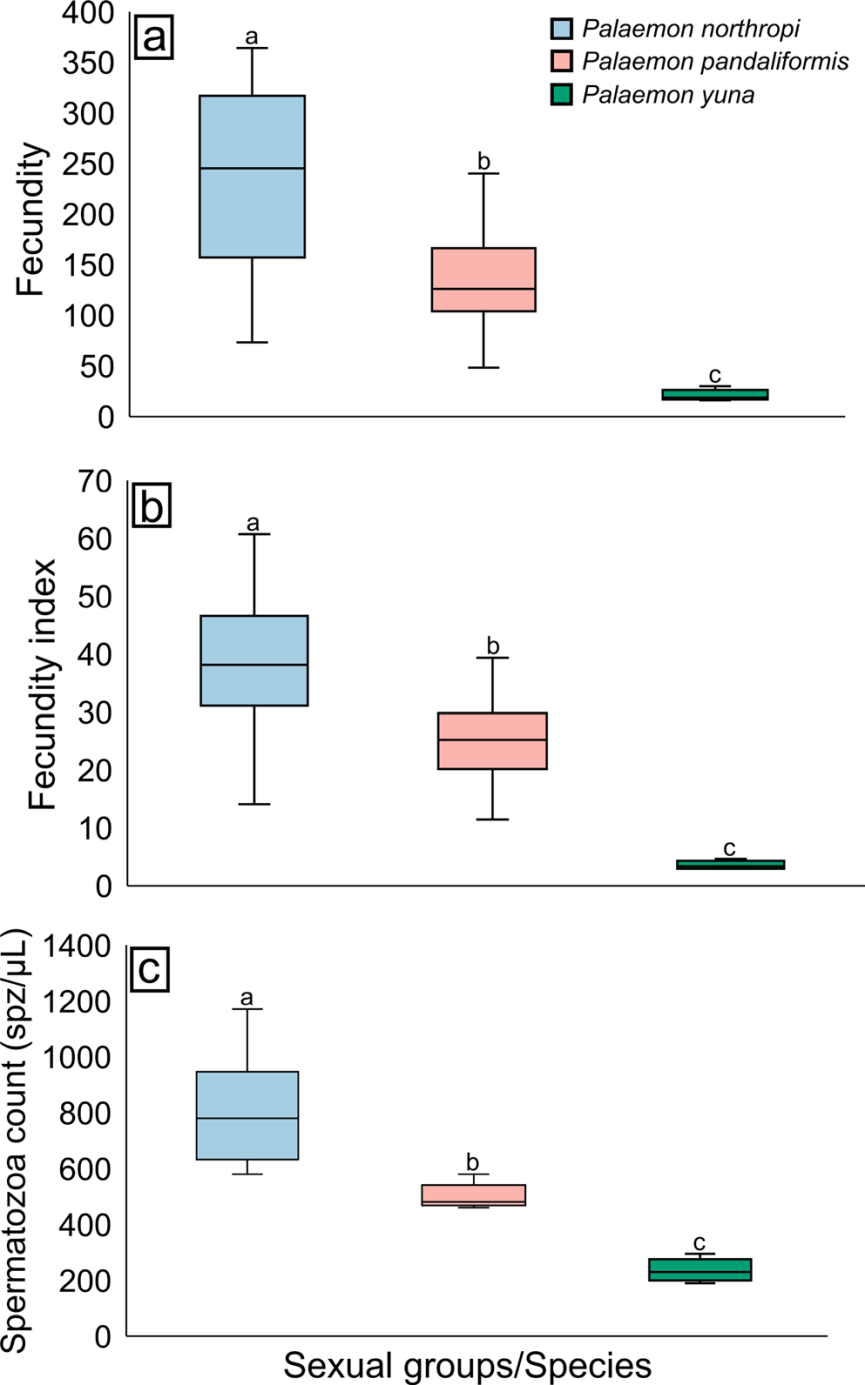
Variation in fecundity and spermatozoa count among *Palaemon* spp. **a** Mean fecundity of females of each species. *Palaemon northropi* showed the highest fecundity, whereas *P. yuna* exhibited the lowest. **b** Fecundity index of females of each species, following the same pattern observed for mean fecundity. **c** Spermatozoa count of males of each species. Males of *P. northropi* show the highest spermatozoa concentration, while those of *P. yuna* show the lowest. Different letters above the boxplots indicate significant differences among the compared groups (*p* < 0.05)

Overall, the same pattern was observed for both mean fecundity and the fecundity index, following the order: *P. northropi* > *P. pandaliformis* > *P. yuna*. Mean fecundity was 257 ± 133 embryos in *P. northropi*, 136 ± 43 embryos in *P. pandaliformis*, and 21 ± 6 embryos in *P. yuna*. The fecundity index showed a similar pattern, with values of 41 ± 17 embryos in *P. northropi*, 26 ± 6 embryos in *P. pandaliformis*, and 4 ± 1 embryos in *P. yuna*. Statistical differences were detected for both mean fecundity (Kruskal-Wallis, H = 25.58, *p* < 0.001) and the fecundity index (Kruskal-Wallis, H = 25.43, *p* < 0.001) among all *Palaemon* species (Dunn’s test, *p* < 0.01) (Fig. 3A, B; Table 1).

Regarding spermatozoa count, a pattern similar to that observed for fecundity was identified, with *P. northropi* showing the highest spermatozoa concentration and *P. yuna* the lowest, i.e., *P. northropi* > *P. pandaliformis* > *P. yuna*. Mean spermatozoa count was 805 ± 208 spz/µL in *P. northropi*, 499 ± 47 spz/µL in *P. pandaliformis*, and 236 ± 41 spz/µL in *P. yuna*. Significant differences were detected among the species (ANOVA, F = 24.93, *p* < 0.001). Spermatozoa count in *P. northropi* was significantly higher than in *P. pandaliformis* (Tukey’s test, *p* = 0.006) and *P. yuna* (Tukey’s test, *p* < 0.001). Additionally, spermatozoa count in *P. pandaliformis* also differed significantly from that observed in *P. yuna* (Tukey’s test, *p* = 0.02) (Fig. 3C; Table 1).

### Embryos and spermatozoa size

**Fig. 4:**
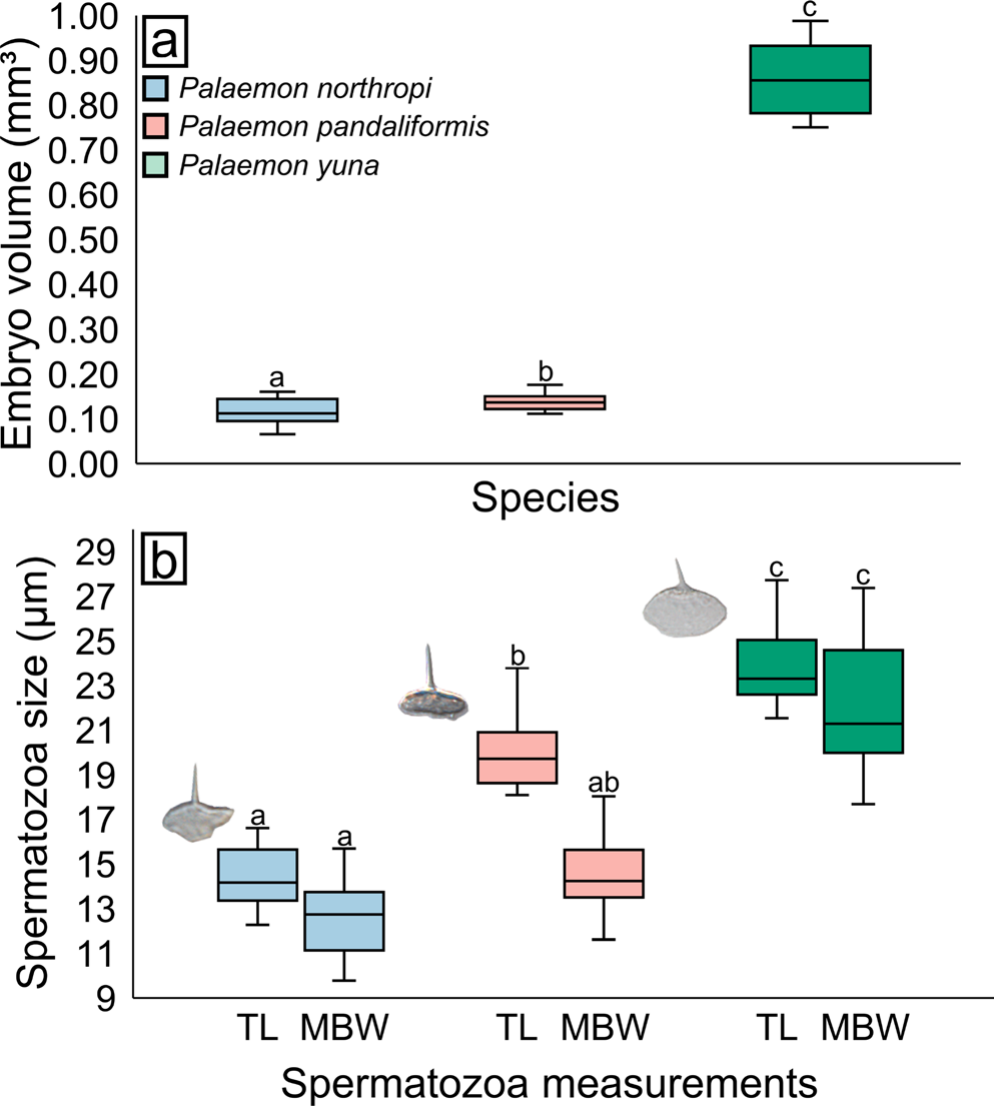
Variation in embryo volume and spermatozoa size in *Palaemon* spp. **a** Variation in embryo volume. Embryos of *P. yuna* are the largest among the three species, followed by those of *P. pandaliformis* and *P. northropi*. **b** Variation in spermatozoa size; both total length (TL) and main body width (MBW) are greater in *P. yuna*. Next to the boxplots, spermatozoa of the three species are illustrated: *P. northropi* – TL: 15.75 μm and MBW: 13.39 μm; *P. pandaliformis* – TL: 18.08 μm and MBW: 15.5 μm; and *P. yuna* – TL: 26.83 μm and MBW: 27.22 μm. Different letters above the boxplots indicate significant differences among the compared groups (*p* < 0.05)

*Palaemon northropi* had the smallest embryo volume among the species analyzed, whereas *P. yuna* exhibited the largest embryos (i.e., *P. yuna* > *P. pandaliformis* > *P. northropi*). Mean embryo volume was 0.11 ± 0.03 mm³ in *P. northropi*, 0.14 ± 0.02 mm³ in *P. pandaliformis*, and 0.86 ± 0.09 mm³ in *P. yuna*. Significant differences were detected among all species (Kruskal-Wallis, H = 19.4, *p* < 0.001). *Palaemon northropi* had embryos significantly smaller than those of *P. pandaliformis* (Dunn’s test, *p* = 0.04) and *P. yuna* (Dunn’s test, *p* < 0.001), and *P. pandaliformis* had embryos significantly smaller than *P. yuna* (Dunn’s test, *p* = 0.001) (Fig. 4A; Table 1).

The morphometric analyses of spermatozoa revealed a pattern similar to that observed for embryo size, with *P. northropi* exhibiting the smallest spermatozoa and *P. yuna* the largest (i.e., *P. yuna* > *P. pandaliformis* > *P. northropi*). In *P. northropi*, TL and MBW measured 14.35 ± 1.31 μm and 12.65 ± 1.79 μm, respectively. For *P. pandaliformis*, these values were 20.03 ± 1.56 μm (TL) and 14.53 ± 1.31 μm (MBW), whereas spermatozoa of *P. yuna* exhibited a TL of 23.8 ± 1.6 μm and an MBW of 21.91 ± 2.7 μm.

Significant differences were detected for both TL (Kruskal-Wallis, H = 50.57, *p* < 0.001) and MBW (Kruskal-Wallis, H = 42.9, *p* < 0.001). For TL, all pairwise comparisons among species were significant (Dunn’s test, *p* < 0.001). For MBW, significant differences were observed for most comparisons, except between *P. northropi* and *P. pandaliformis* (Dunn’s test, *p* = 0.05). All other comparisons showed significant differences (Dunn’s test, *p* < 0.001) (Fig. 4B; Table 1).

Overall, embryo volume in *Palaemon* females showed a strong positive correlation with spermatozoa size in males, indicating that gamete size increased in parallel with embryo size (*P. northropi*: *r* = 0.98, *p* < 0.001; *P. pandaliformis*: *r* = 0.97, *p* < 0.001; *P. yuna*: *r* = 0.94, *p* < 0.001).

### Reproductive output, per-offspring investment, and weapon investment

**Fig. 5:**
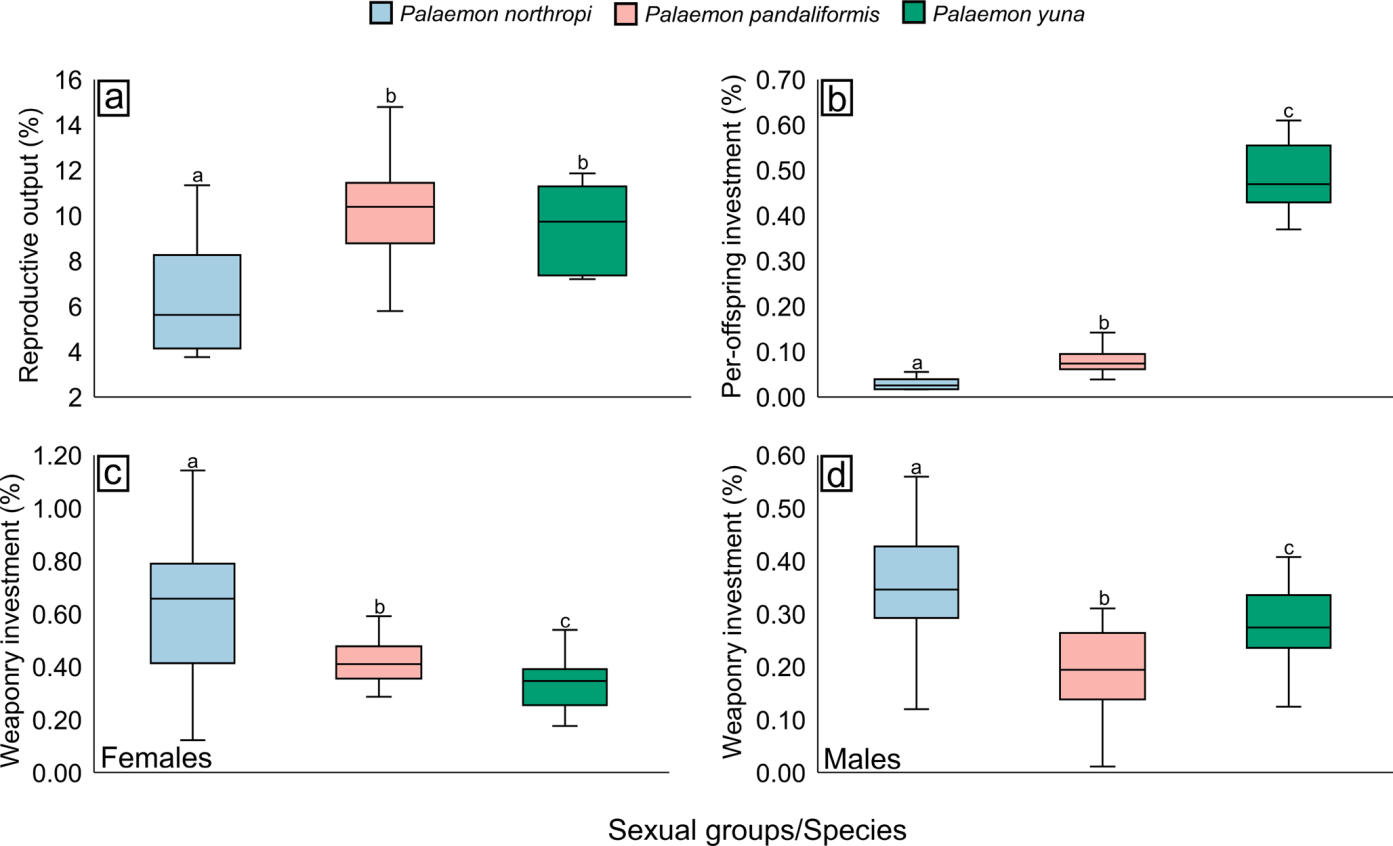
Reproductive output, per-offspring investment, and weapon investment in *Palaemon* spp. **a** Reproductive output among females of the three species. *P. pandaliformis* and *P. yuna* allocate more energy to reproduction. **b** Per-offspring investment among females of the three species. *P. yuna* directs a substantially greater amount of energy to each embryo compared with the other species. **c**, **d** Weapon investment in females and males, respectively. *Palaemon northropi* is the species that allocates the most energy to cheliped development, regardless of sex. Different letters above the boxplots indicate significant differences among the compared groups (*p* < 0.05)

Ovigerous females of *P. northropi* showed a mean RO of 6.34 ± 2.49%, whereas *P. pandaliformis* exhibited an RO of 10.01 ± 2.30% and *P. yuna* of 9.50 ± 1.93%. Significant differences were detected among species (Kruskal–Wallis, H = 14.9, *p* < 0.001). The RO of *P. northropi* was significantly lower than that of *P. pandaliformis* (Dunn’s test, *p* < 0.001) and *P. yuna* (Dunn’s test, *p* = 0.03). In contrast, no statistical difference was found between *P. pandaliformis* and *P. yuna* (Dunn’s test, *p* = 0.68) (Fig. 5A; Table 1).

Regarding per-offspring investment, *P. northropi* exhibited a mean value of 0.03 ± 0.01% per embryo, *P. pandaliformis* of 0.08 ± 0.03%, and *P. yuna* of 0.48 ± 0.08%. Significant differences were detected in all pairwise comparisons among species (Kruskal–Wallis, H = 34.91, *p* < 0.001; Dunn’s test, *p* < 0.01) (Fig. 5B; Table 1).

Regarding weaponry investment, females of *P. northropi* exhibited a mean value of 0.64 ± 0.29%, whereas females of *P. pandaliformis* showed 0.43 ± 0.10%, and females of *P. yuna* 0.34 ± 0.10%. Among males, *P. northropi* displayed a mean investment of 0.41 ± 0.12%, *P. pandaliformis* 0.20 ± 0.07%, and *P. yuna* 0.28 ± 0.07%. Significant differences were recorded both among females (Kruskal–Wallis, H = 27.01, *p* < 0.001; Dunn’s test, *p* < 0.05) and among males of the three species (Kruskal–Wallis, H = 31.89, *p* < 0.001; Dunn’s test, *p* < 0.01), with all pairwise comparisons being significant (Fig. 5C, D; Table 1).

## Discussion

The present study demonstrates that the degree of colonization of freshwater environments is associated with marked variation in reproductive traits, acting as an essential adaptive mechanism for reproductive success in these habitats. Although reproductive adaptations in females have been documented across different groups of decapods (Anger and Moreira 1998; Vogt 2013; Anger 2016; Bauer 2023; Pantaleão et al. 2025), these data have generally been examined in isolation or without an explicit comparative framework focusing on phylogenetically related species occurring in contrasting environmental contexts, such as marine, estuarine, and freshwater systems. By comparing closely related species that differ in life history, habitat use, and the extent of freshwater colonization, our study provides a comparative perspective that allows reproductive traits to be interpreted in an evolutionary and ecological context and highlights clear differences in reproductive investment associated with freshwater colonization. Importantly, these differences are expressed not only in female traits, such as fecundity, embryo size, and per-offspring investment, but also in male reproductive traits, including spermatozoa size and concentration. Moreover, we show that males, a component traditionally understudied in discussions of reproductive adaptation in Caridea, also exhibit reproductive adaptations that align with the patterns observed in females. This finding suggests coordinated adjustments between male and female reproductive traits, likely reflecting shared energetic constraints and coevolutionary interactions between gametes and embryos, rather than independent optimization of each sex.

All three *Palaemon* species analyzed in this study exhibited the same pattern of sexual size dimorphism, wherein females are consistently larger than males. This morphological pattern is characteristic of species exhibiting the pure search mating system, in which smaller males roam the environment to search for receptive females and do not engage in aggressive behaviors or physical contests over mates (Bauer 2023). Available evidence indicates that this mating system is predominant within the genus *Palaemon*, with no records of species in which males are larger than females or exhibit agonistic behaviors associated with copulation, traits that would suggest alternative mating systems involving male–male competition (Berglund 1981; Anger and Moreira 1998; Bauer and Abdalla 2001; Kim 2005; Paschoal et al. 2013; Emmerson et al. 2017; Barros-Alves et al. 2019). The consistency of this pattern across species occupying distinct environments suggests that sexual size dimorphism in *Palaemon* can be evolutionarily conserved, while other reproductive traits, particularly those related to gamete production and offspring provisioning, appear to be more labile and responsive to environmental pressures.

Differences in size variation between females and males of the three species were observed in this study. Among females, although mean CL did not show pronounced variation, significant differences were still detected, with *P. northropi* exhibiting the largest sizes. Among males, the range of mean values was even narrower, with *P. yuna* males being the largest among the analyzed species. Despite the low mean variation among females, it is noteworthy that *P. northropi* can reach maximum sizes exceeding those of *P. pandaliformis* and *P. yuna*, with some females measuring approximately 3 mm more than the largest females of the other two species. This pattern may be associated with differences in the environmental conditions experienced by each species, as marine environments, such as those inhabited by *P. northropi*, are generally characterized by higher and more stable resource availability when compared to freshwater systems, which often impose stronger ecological constraints (Anger 2006; Vogt 2013). Under such conditions, increased food availability may allow marine species to attain larger maximum body sizes, potentially through sustained growth over longer periods. In other words, a higher and/or more stable food supply may enable *P. northropi* to maintain a faster growth rate than its estuarine and freshwater congeners. However, this pattern is not observed in males, which exhibit only slight interspecific variation in body size. This relative uniformity may reflect constraints associated with the mating system rather than environmental effects alone. Since these organisms exhibit a pure search mating system, without aggressive behaviors, territoriality, or male–male competition (Bauer, 2023), there is little selective pressure favoring increased male body size. Consequently, energy that could otherwise be allocated to growth may instead be channeled toward other biological functions.

In the present study, *P. northropi* exhibited the highest mean fecundity, whereas *P. yuna* showed the lowest, a pattern that was also reflected in spermatozoa production. It is well known that the energetic cost associated with the production of yolk-rich oocytes, as observed in *P. yuna*, is high, since these oocytes, later converted into embryos, must sustain prolonged embryonic development (Anger 2001; Vogt 2013). However, comparatively little attention has been given to whether male reproductive traits in decapod crustaceans exhibit parallel shifts to those documented for female oocytes and embryos along environmental gradients, particularly with respect to gamete production and energetic allocation. The results obtained here demonstrate that spermatozoa production varies consistently among species, suggesting that this trait also entails non-negligible energetic costs and that males, similarly to females, adjust their reproductive investment in response to a reduced number of eggs available for fertilization. Thus, our findings indicate that freshwater colonization is associated with coordinated shifts in both female and male reproductive traits, rather than affecting female traits alone. This pattern reinforces the idea that multiple components of reproduction may evolve in an integrated manner, reflecting shared energetic constraints and life-history trade-offs, as previously noted for other animal groups (Fischer et al. 2009; Immler et al. 2011; Soulsbury and Iossa 2024).

Similarly to the pattern observed for fecundity and spermatozoa concentration, embryo and spermatozoa size, here represented by embryo volume and male gamete length and width, also followed the same interspecific gradient. *Palaemon yuna* exhibited the largest embryos and spermatozoa, whereas *P. northropi* displayed the smallest, corresponding, respectively, to the strictly freshwater and marine species. This correspondence reinforces the association between the degree of freshwater dependence and increased per-gamete investment, rather than indicating discrete, habitat-specific shifts. Equivalent patterns have been described for female and male gametes in other invertebrates, such as flies and butterflies, in which these associations have been suggested to reflect shared genetic or developmental constraints, including pleiotropic effects on gamete size (Pitnick et al. 2003; Fischer et al. 2009; Soulsbury and Iossa 2024). Accordingly, selective pressures acting on the gametes of one sex may also influence reproductive traits of the opposite sex (Pitnick et al. 2009), resulting in correlated evolutionary responses rather than independent optimization of male and female traits. In the specific case of the species analyzed here, *P. yuna* produces embryos with volumes nearly eight times larger than those of *P. northropi*. Among males, this difference is less pronounced, with spermatozoa of *P. yuna* being approximately twice as large as those of *P. northropi*. Even so, these results suggest that increases in gamete size may occur in a coordinated, albeit asymmetric, manner between the sexes, reflecting a shared set of life-history trade-offs and energetic constraints rather than identical scaling between male and female gametes.

The patterns observed showed that reproductive output differs markedly among the three species analyzed. Although *P. yuna* and *P. pandaliformis* exhibit similar total reproductive output, both higher than that of *P. northropi*, the per-offspring analysis reveals that *P. yuna* allocates far more energy to each embryo. This result reflects the typical trade-off of abbreviated larval development, in which few larvae hatch in a highly advanced state, with substantial yolk reserves and a shortened period of exposure in the water column, thereby maximizing survival in low-productivity freshwater environments where larval retention is crucial (Anger 2001; Vogt 2013). In contrast, *P. pandaliformis* and *P. northropi* invest energy in producing large numbers of small embryos, characteristic of extended larval development (Moura et al. 1990; Gamba 1998). In these species, larvae hatch in a poorly developed state, depend on external feeding, and remain exposed to predation for long periods, resulting in a lower probability of reaching the juvenile stage (Anger 2001).Thus, the differences observed reflect contrasting reproductive strategies shaped by environmental conditions and by the challenges imposed on larval dispersal and survival in each habitat. In a particularly illustrative way, the estuarine species *P. pandaliformis*, which inhabits an environment transitional in salinity, stability, and resource availability, exhibits an intermediate pattern, combining reproductive traits more similar to those of the marine species, such as the high production of small eggs, with others more comparable to the freshwater species, such as the higher total reproductive output.

The weaponry investment by the species analyzed here further corroborate patterns previously documented for the group. Females allocate nearly twice as much energy to the development of the chelipeds compared to males, reinforcing the pure search mating system proposed as typical for these species. Despite this, even among females, mean weaponry investment does not reach 1% of body mass, indicating that these structures remain relatively small. Nevertheless, chelipeds are used by females in a wide variety of tasks, including agonistic behaviors related to defending resources such as shelter or food (Rapparport and Lord 2021). Although such interactions occur, they do not escalate into intense aggressive disputes, thereby limiting selective pressure for exaggerated weapon development, in contrast to what has been documented for other caridean shrimps with different mating systems (Bauer 2023).

Taken together, our results demonstrate that colonization of freshwater environments promotes a coordinated suite of shifts in the reproductive traits of the species analyzed here, affecting both females and males. These shifts include adjustments in the number and size of gametes, fecundity rates, and the allocation of energy among offspring. The convergence of these patterns points to integrated responses to energetic and ecological constraints imposed by freshwater habitats, reinforcing that reproductive evolution in these systems does not occur independently between the sexes but instead likely reflects shared selective pressures. By revealing how multiple components of reproduction shift jointly during the transition to freshwater environments, this study provides broader insights into the mechanisms through which environmental gradients shaped diversification and the life-history evolution.

## Data Availability

No datasets were generated or analyzed during the current study.
